# Changing Epidemiology of TB in Shandong, China Driven by Demographic Changes

**DOI:** 10.3389/fmed.2022.810382

**Published:** 2022-03-09

**Authors:** Qianying Lin, Sourya Shrestha, Shi Zhao, Alice P. Y. Chiu, Yao Liu, Chunbao Yu, Ningning Tao, Yifan Li, Yang Shao, Daihai He, Huaichen Li

**Affiliations:** ^1^Department of Applied Mathematics, Hong Kong Polytechnic University, Kowloon, Hong Kong SAR, China; ^2^Michigan Institute for Data Science, University of Michigan, Ann Arbor, MI, United States; ^3^Bloomberg School of Public Health, Johns Hopkins University, Baltimore, MD, United States; ^4^JC School of Public Health and Primary Care, Chinese University of Hong Kong, Johns Hopkins University, Shatin, Hong Kong SAR, China; ^5^Shandong Provincial Hospital Affiliated to Shandong University, Jinan, China; ^6^Shandong Chest Hospital, Jinan, China; ^7^Department of Respiratory and Critical Care Medicine, Shandong Provincial Hospital Affiliated to Shandong First Medical University, Jinan, China

**Keywords:** Tuberculosis, heterogeneity, urbanization, aging, age-period-cohort model

## Abstract

Tuberculosis (TB) incidence has been in steady decline in China over the last few decades. However, ongoing demographic transition, fueled by aging, and massive internal migration could have important implications for TB control in the future. We collated data on TB notification, demography, and drug resistance between 2004 and 2017 across seven cities in Shandong, the second most populous province in China. Using these data, and age-period-cohort models, we (i) quantified heterogeneities in TB incidence across cities, by age, sex, resident status, and occupation and (ii) projected future trends in TB incidence, including drug-resistant TB (DR-TB). Between 2006 and 2017, we observed (i) substantial variability in the rates of annual change in TB incidence across cities, from -4.84 to 1.52%; (ii) heterogeneities in the increments in the proportion of patients over 60 among reported TB cases differs from 2 to 13%, and from 0 to 17% for women; (iii) huge differences across cities in the annual growths in TB notification rates among migrant population between 2007 and 2017, from 2.81 cases per 100K migrants per year in Jinan to 22.11 cases per 100K migrants per year in Liaocheng, with drastically increasing burden of TB cases from farmers; and (iv) moderate and stable increase in the notification rates of DR-TB in the province. All of these trends were projected to continue over the next decade, increasing heterogeneities in TB incidence across cities and between populations. To sustain declines in TB incidence and to prevent an increase in Multiple DR-TB (MDR-TB) in the future in China, future TB control strategies may (i) need to be tailored to local demography, (ii) prioritize key populations, such as elderly and internal migrants, and (iii) enhance DR-TB surveillance.

## 1. Introduction

Tuberculosis (TB) remains the largest single-agent infectious source of mortality worldwide, with 1.4 million deaths in 2019 (1). As one of the most urgent epidemic goals, the World Health Organization (WHO) End TB Strategy aims to reduce the TB incidence worldwide in 2035 to 90% of the 2015 levels and to eradicate TB by 2050 (2). With the second largest TB burden after India (1), China constitutes approximately 0.9 million TB infections every year; therefore, sustaining declines in TB in China is essential to achieve global goals. Despite considerable success in reducing the growth of TB in China, the midterm goal of 50% reduction in incidence rate by 2025 compared to 2015 is not likely to be met, given around 10.8% reductions between 2015 and 2019 (2, 3).

Furthermore, China is undergoing a massive demographic transition, which will have important implications for TB epidemiology and control. First, the proportion of the population older than 65 years, increased from 7.9% in 2006 to 12.6% in 2019 in China (4), and is projected to increase to 27.9% by 2050 (5). Along with the increase of the elderly population, studies have also reported increased burden of TB in this population (6), indicating that reactivation of remote infection is an increasing important driver of TB in China (7). Second, China has been experiencing high rates of urbanization, driven by internal migration of population from rural counties, towns, and cities to large urban centers (8, 9). Internal migration can have important implications for TB transmission and control. Migrants are more likely to experience poor living conditions (i.e.,overcrowding or poor sanitation), belong to lower socio-economic status, and face delays in TB diagnosis and care, making them more vulnerable to TB (10). These factors can increase differences in TB epidemiology between cities, and within populations in the cities. Therefore, a thorough understanding of the role of these demographic factors on driving heterogeneities in TB epidemiology can inform future TB control strategies. There is also increasing concern of drug-resistant TB (DR-TB) in China, given that it makes up about a third of all DR-TB in the world, and studies have noted increase in Multiple DR-TB (MDR-TB) notification rates in the recent decade (6, 11).

Shandong Province, the focus of this study, is located in eastern China and has the second-largest population (more than one billion) in China, of which 15.8% were over 65 years old, and 50.4% were from rural areas in 2019 (12). The Fifth National TB Epidemiological Survey reported 211,900 TB infections in Shandong in 2010, of which 66.7% were residents of rural areas (13). With prevention and control efforts, the incidence rate dropped from 40.89/100,000 in 2011 to 32.7/100,000 in 2016 and 25.93/100,000 in 2020 in Shandong (14, 15), while the rate of DR-TB and MDR-TB were relatively high between 2007 and 2014 (16). Using local data on TB notification and demography, along with age-period-cohort models, we investigated how demographic changes in the last two decades have affected local TB epidemiology across the cities and projected how these changes were likely to be accentuated in the next decade.

## 2. Materials and Methods

### 2.1. Data

We compiled TB notification data between 2006–2017 from the Tuberculosis Information Management System, Chinese Center for Disease Control and Prevention (CDC) (17), from seven cities in Shandong, namely, Jinan, Yantai, Weifang, Jining, Linyi, Dezhou, and Liaocheng. Notification data included information on sex, age, date of birth, occupation, resident status, and date when diagnosis was made (Supplementary Table S1). We also collated data on DR-TB from laboratory-confirmed records from TB Surveillance System (17) for four commonly used drugs, isoniazid (INH), rifampicin (RFP), ethambutol (EMB), and streptomycin (SM), from 38 TB monitoring units, including two province-level hospitals, 21 county-level hospitals, and 13 municipal-level local health departments, out of 142 such units across Shandong (Supplementary Table S2). Population demography data were taken from the Shandong Statistics Yearbook (12) and The Sixth National Population Census of 2010 (18). A summary of population and TB incidence across all seven cities can be found in Table 1.

**Table 1:** Population size and growth, and TB incidence and change across 7 cities.

	**Jinan**	**Yantai**	**Linyi**	**Liaocheng**	**Weifang**	**Jining**	**Dezhou**
TB incidence							
2006	1775	2471	5569	2475	2817	3512	2858
2017	1706	1633	4112	2927	2382	1873	1197
Changes/yr (%)	-0.32	-2.83	-2.18	+1.52	-1.29	-3.89	-4.84
Total population (100K)							
2006	60.335	64.998	102.273	57.282	85.529	81.183	55.785
2017	64.362	65.423	116.190	63.969	90.801	88.321	59.535
Changes/yr (%)	+0.56	+0.05	+1.13	+0.97	+0.51	+0.73	+0.56
Female population (100K)							
2006	30.063	32.319	49.988	28.332	42.328	39.643	27.585
	(49.83%)	(49.72%)	(48.88%)	(49.46%)	(49.49%)	(48.83%)	(49.45%)
2017	32.441	32.852	55.992	31.050	45.002	42.737	29.348
	(50.40%)	(50.21%)	(48.19%)	(48.54%)	(49.56%)	(48.39%)	(49.30%)
Changes/yr (%)	+0.65	+0.14	+1.00	+0.80	+0.53	+0.65	+0.53
60+ population (100K)							
2006	8.061	10.308	12.876	7.413	12.058	13.120	7.282
	(13.36%)	(15.86%)	(12.94%)	(12.59%)	(14.10%)	(16.16%)	(13.05%)
2017	13.777	16.485	21.063	11.288	19.590	16.046	10.145
	(21.41%)	(25.20%)	(18.13%)	(17.65%)	(21.57%)	(18.17%)	(17.04%)
Changes/yr (%)	+5.91	+4.99	+5.30	+4.36	+5.21	+1.86	+3.28
Migrants (100K)							
2007	9.602	2.944	1.503	0.682	2.582	1.503	0.696
2017	24.693	10.207	5.776	2.145	8.554	5.776	4.525
Changes/yr (%)	+14.29	+22.43	+25.85	+19.50	+21.03	+25.85	+50.01

### 2.2. Age-Period-Cohort Model

Age, birth cohort, and calendar period of diagnosis are three risk factors and time scales that are important in epidemiological risk analyses. However, the sole impacts of these three time scales are unidentifiable due to the linear relation among them, i.e.,age + cohort = period (19). To overcome this difficulty, Kuang et al. proposed a canonical parameterization approach to map the original variable space into a second-order difference of the original variables space and a linear plane determined by three points of the original variable space (to reduce 4 degrees of freedom) (20). Forecasts are conducted in steps: extracting and estimating all effects from the time series, extrapolating the period effect, and forecasting the time series by combining all three effects (21). In addition to chronic diseases, age-period-cohort (APC) models are also applicable to infectious diseases such as TB and AIDS (22), given that these infectious diseases share a long-run infection process.

Throughout this study, we use the following notations and classifications: TB notification rate (i.e., the number of notifications per 100 thousand population), DR-TB tested notification rate (i.e., the number of notifications per 100 tested notifications), DS-TB (drug-sensitive TB, i.e., sensitive to INH, RFP, EMB, and SM), INH-resistant, and RFP-resistant. To distinguish from TB notification rate, we use the term *proportion* to indicate the fraction of certain demographic groups among all TB notifications.

We fitted APC models to the TB notifications and DR-TB notifications separately and estimated the individual effect of age, birth cohort and period on TB notification rate and DR-TB tested notification rate. We conducted the Poisson bootstrap simulation method 100 times to explore model sensitivity (23).

Several elements and assumptions were needed for projections: (1) a predicted population for both sexes by multiplying the average change rate from 2006–2017 across the seven cities, as well as the total future population in these seven cities; (2) the forecast of TB notifications for both sexes across seven cities and the total TB notifications in these seven cities; (3)the forecast of notified DR-TB rates from total laboratory-confirmed TB notifications in 38 units; (4) the TB notification rate (i.e.,$\frac{\text{TB notifications}}{\text{100K population}}$) estimated by TB notified cases from the seven cities is representative across the province; (5) the DR-TB tested rate (i.e.,$\frac{\text{DR-TB notifications}}{\text{100 TB notifications}}$) estimated by records from 38 units is representative across the 142 units; (6) DR-TB laboratory-confirmed ratio was considered at 50, 75, and 100% (24).

Therefore, by combining (1) and (2) using methods described in Kuang et al. (21), we conducted a 10-year forecast of TB notification rate for both sexes across seven cities. The 6-year forecasts of DR-TB rates [i.e.,(3)] for INH-resistant cases, RFP-resistant cases and MDR-TB cases were estimated with methods in Kuang et al. (21). All the estimated elements and assumptions mentioned above resulted in a rough forecast of DR-TB rates (i.e.,$\frac{\text{DR-TB notifications}}{\text{100K population}}$) under three laboratory-confirmed ratios in Shandong.

All analyses, data visualizations, and models were performed and constructed in R (version 3.4.3). In particular, the R package “apc” was implemented for fitting APC models and forecasting future trends (25). Using APC model, we estimated the patterns of age effects, period effects, and cohort effects, as shown in Supplementary Material.

## 3. Results

### 3.1. Historical Trends of TB Incidence Across Cities

Table 1 summarizes TB incidence and its annual changes across seven cities, along with related demographic information, between 2006 and 2017. The considerable variability in the annual reduction rate of TB incidence across cities was observed, from -4.84%/yr in Dezhou to +1.52%/yr in Liaocheng. In the context of continuous population growth from 2006 to 2017, we observed a consistent and drastic increase in the proportion of the population over 60 years old and the migrant population across all seven cities. Meanwhile, though the female population increased from 2006 and 2017, the difference in the change of its percentage across seven cities is shown: increase in Jinan, Yantai, Weifang, and decrease in Linyi, Liaocheng, Jining, and Dezhou.

**Figure 1:**
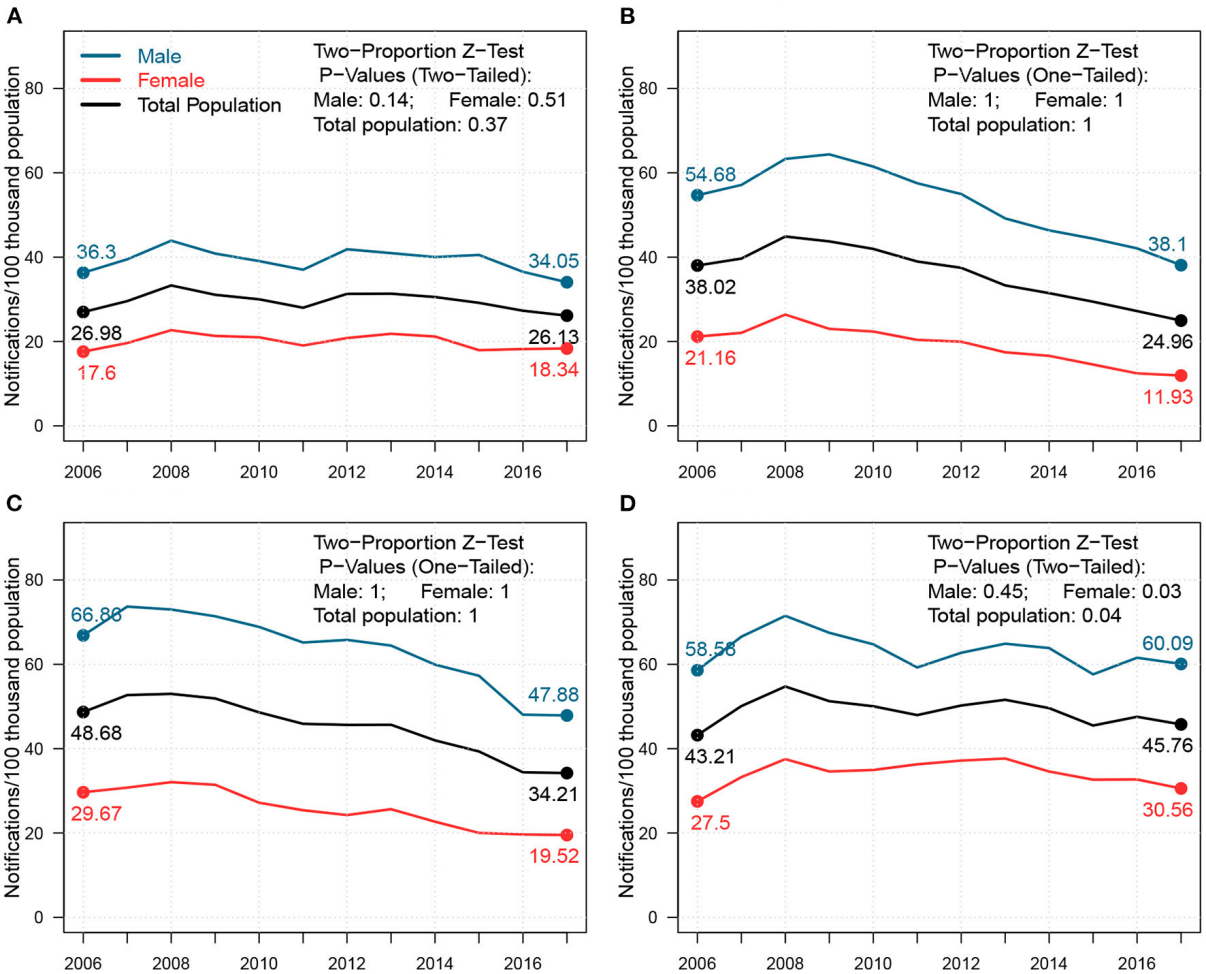
Changes in the notification rates for males, females, and total population from 2006–2017 in **(A)** Jinan, **(B)** Yantai, **(C)** Linyi, and **(D)** Liaocheng. In each panel, the blue, red, and black lines represent notification rate trends; the P-Values of the Two-Proportion Z-Test for males, females, and total population in each cities are shown in the top-right corner.

Heterogeneity in TB notification rates was observed between males and females in Shandong (Figure 1). The notification rates of males were higher than those of females throughout the 12 study years. However, heterogeneities in gaps between male and female cases were observed between cities. TB notification rates in Yantai and Linyi for both males and females significantly declined from 2006–2017, where p-values of one-tailed Two-Proportion Z-test (26) are 1.00 for both males and females in both Yantai and Linyi. However, those in Jinan and Liaocheng remained relatively steady, where p-values of Two-Tailed Two-Proportion Z-Test were 0.14 and 0.51 for males and females in Jinan, and 0.45 and 0.03 for males and females in Liaocheng, respectively. Although stable, Jinan experienced a slight reduction in the TB notification rate for males and a slight increase for females. TB notifications for both males and females in Liaocheng increased from 2006–2017.

### 3.2. The “Aging” Phenomenon Among TB Notifications

**Figure 2:**
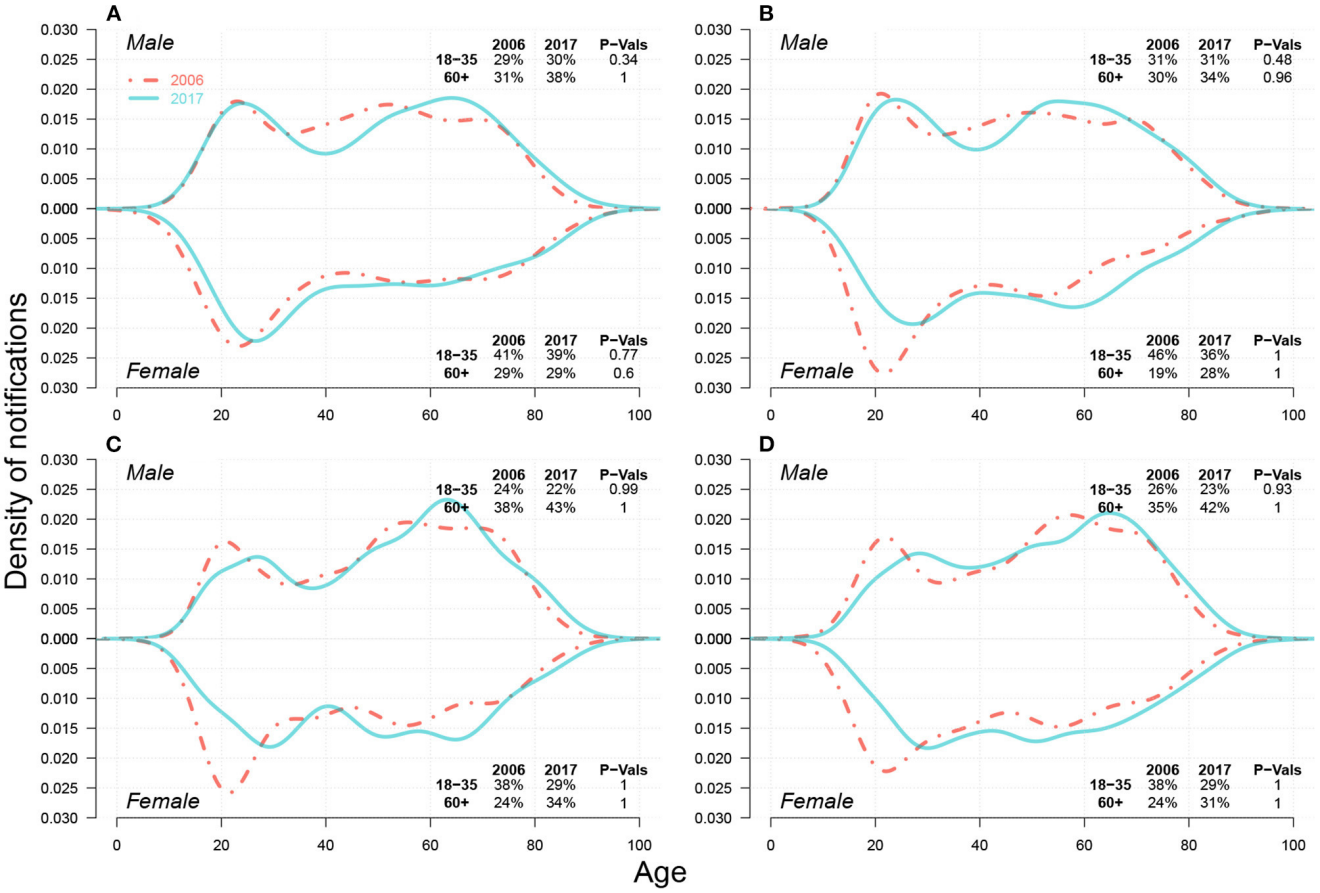
Case density changes in 2006 and 2017 for males (upper subpanels) and females (lower subpanels) in **(A)** Jinan, **(B)** Yantai, **(C)** Linyi, and **(D)** Liaocheng. In each panel, red dashed-and-dotted and green solid lines represent densities in 2006 and 2017, respectively. We show the changes in case *proportions* (%) of young adults (18–35) and seniors (60+) in 2006 and 2017 in the top-right corner for males and in the bottom-right corner for females; the corresponding P-Values for the decrease in case proportion for young adults and the increase in that for seniors between 2006 and 2017 *via* One-sided Two-Proportion Z Tests are also demonstrated, respectively.

In Figure 2, we presented the changes in TB notification density by age between 2006 and 2017 for both sexes across four cities in Shandong. We further highlighted the density changes for young adults (i.e.,18–35) and seniors (i.e.,60+). Homogeneities were present in 2006 across all four cities. For males, young adults and seniors were the two primary sources for TB notifications. For females, TB notifications from young adults occupied a majority of cases. In 2017, however, notification densities by age shifted, and heterogeneity across cities was observed. For males, the *proportion* of young adults mainly remained stable in Jinan and Yantai, and decreased slightly in Linyi and Liaocheng from 2006–2017, while that of seniors increased slightly in all four cities. For females, both *proportions* in Jinan remained unchanged, while large proportions of TB notifications shifted from young adults to seniors in Yantai, Linyi, and Liaocheng. This drastic “aging” phenomenon presented in TB notifications among females from 2006–2017 can be a signal of the increasing burden of reactivated TB (7).

### 3.3. Heterogeneity in TB by Resident Status and Occupation

**Figure 3:**
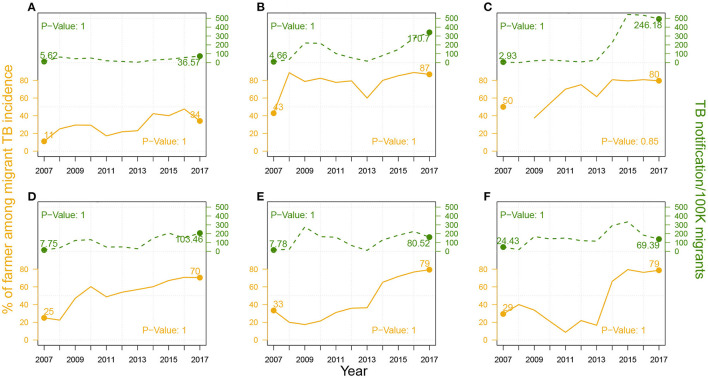
The proportion (%) of farmer cases among migrant TB cases (in solid brown line) and TB notification rates among migrants (in dashed green line) from 2007–2017 in **(A)** Jinan, **(B)** Linyi, and **(C)** Liaocheng, **(D)** Weifang, **(E)** Jining, and **(F)** Dezhou. P-Values of One-sided Two-Proportion Z-Tests for significant increase between 2007 and 2017 in proportion of farmer cases and in TB notification rate among migrants are listed in the bottom-right corner and the top-left corner, respectively.

In the upper part of each panel in Figure 3, we presented the TB notification rates among migrants from 2007 to 2017 in Jinan, Linyi, Liaocheng, Weifang, Jining, and Dezhou. We observed a massive diversity in the notification rates across six cities by 2017, from 36.57 cases per 100 K migrants in Jinan to 170.7 cases per 100 K migrants in Linyi, and around 250 cases per 100 K migrants in Liaocheng. The growth rates of TB notifications among migrants also hugely differed, from 2.81 cases per 100 K migrants per year in Jinan to 15.09 cases per 100 K migrants per year in Linyi and 22.11 cases per 100K migrants per year in Liaocheng. Rapid urbanization rates in 2007–2017 in Linyi (from 38.23 to 57.40%) and Liaocheng (from 33.71 to 50.34%) (12) may have resulted in TB prevalence among migrants. We further investigated components of migrant TB notifications, shown in Supplementary Figure S5, and found that most migrant TB cases in Liaocheng were farmers, for both young adults and seniors, and both males and females.

As demonstrated in Supplementary Figure S6 and Supplementary Table S1, farmer cases represented a majority of migrant cases. We then further explored the changes in the proportion of farmer cases over migrant cases from 2007 to 2017, and the results were shown in the lower part of each panel in Figure 3. By 2017, over 70% of migrant cases were contributed by farmer cases in Linyi, Liaocheng, Weifang, Jining, and Dezhou, and this proportion is generally increasing during the study period.

### 3.4. Projections

**Figure 4:**
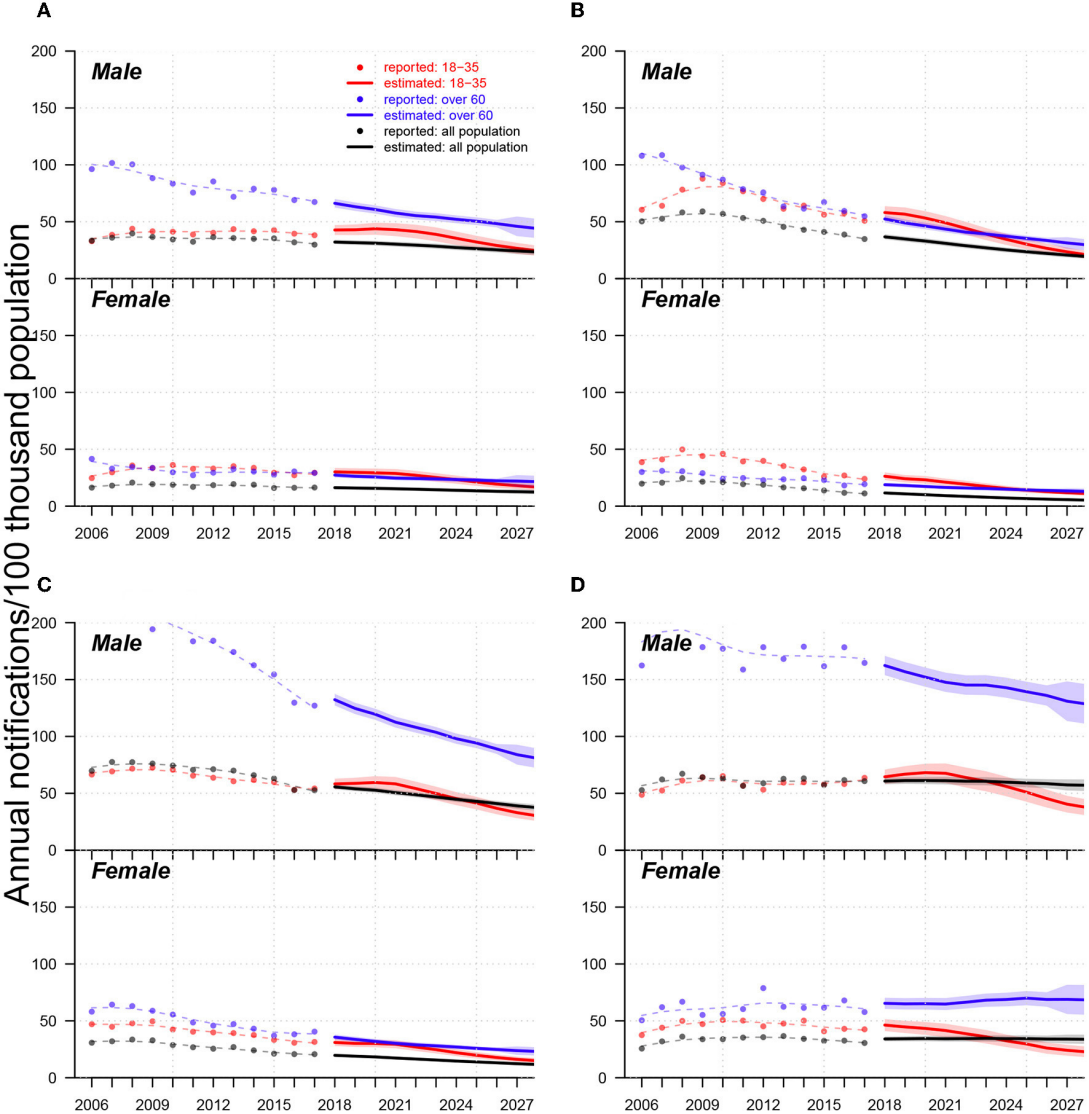
Trends of TB notification rates from 2006-2017 and forecasts from 2018 to 2027 in **(A)** Jinan, **(B)** Yantai, **(C)** Linyi, and **(D)** Liaocheng. In each panel, (i) upper and low subpanels show trends for males and females, respectively; (ii) dots, lines, and shades colored in red, blue, and black indicate reported trends, forecast trends, and 95% confidential intervals for forecasts of annual TB notifications (in the 100 thousand population) for populations aged 18-35 and over 60 years and the total population, respectively; (iii) red, blue, and black dashed lines are smoothed splines that indicate the trends of annual reported TB notifications for the populations aged 18–35 and over 60 years and the total population, respectively.

In Figure 4, regarding overall future trends for males, we predicted that annual TB notifications per 100 K population in 2027 will be approximately 50 in Jinan, Linyi, and Liaocheng and approximately 30 in Yantai. At the end of 2027, the TB notification rates of senior males will be two times greater than those of young adults in Jinan and Yantai and 3–4 times greater than those of young males in Linyi and Liaocheng. For females, the TB notification rate gaps between young adults and seniors will be negligible in Jinan and Yantai, but seniors' notifications will be approximately 2–3 times greater than those of young adults' notifications in Linyi and Liaocheng. We also observed heterogeneities in the leading age groups between two city categories: in Jinan and Yantai, TB notifications from young adults mainly predominate in early years (i.e.,2006–2023), and those from seniors dominate later on; however, in Linyi and Liaocheng, seniors were and were predicted to always be the primary source of TB notifications from 2006–2027. In Liaocheng, unlike in the other three cities, the overall TB notifications per 100 thousand population for males and females were predicted to remain stable at approximately 50 and 30, respectively, and senior female cases were predicted to not decrease in the next ten years.

### 3.5. Trends in DR-TB

**Figure 5:**
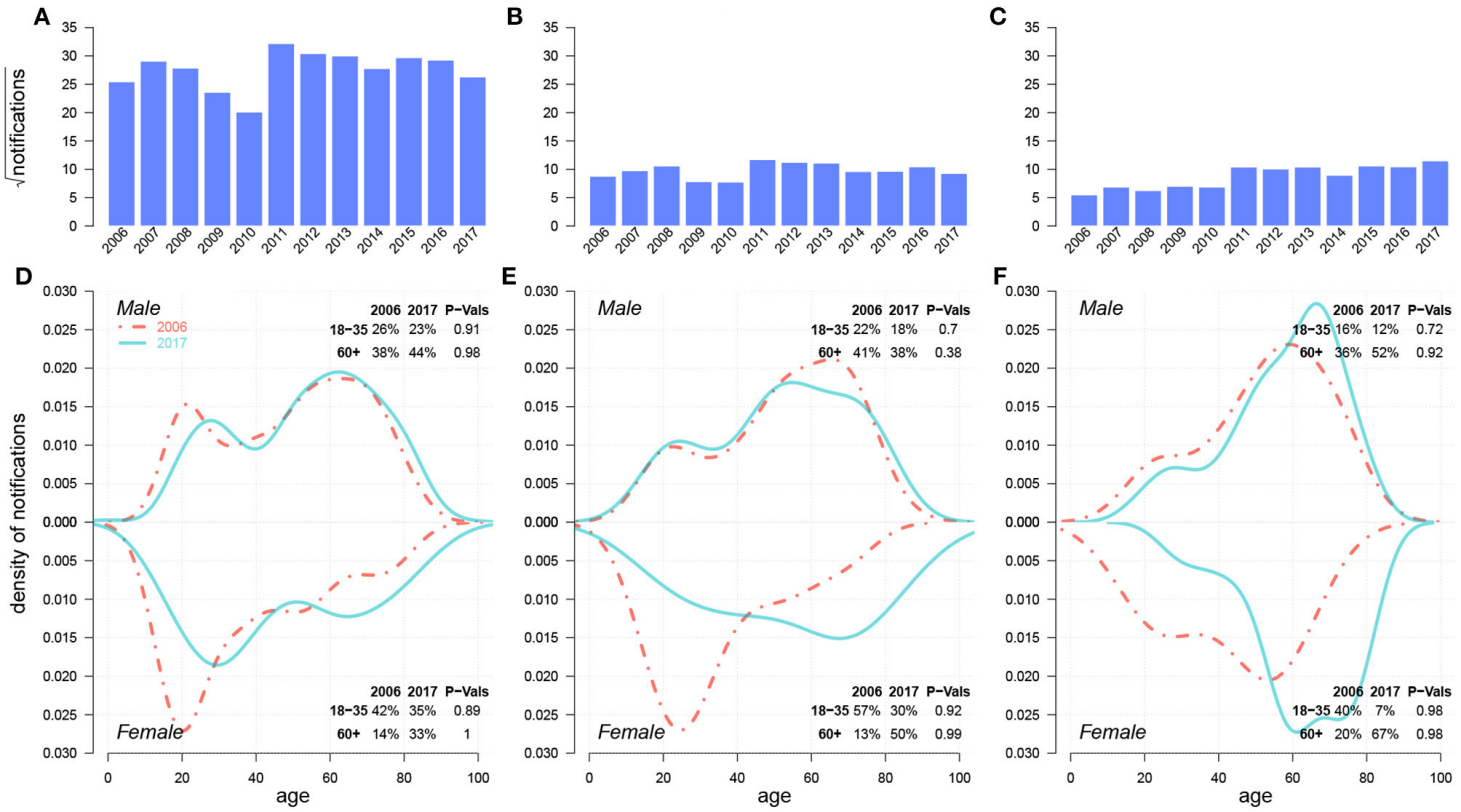
Historical trends from 2006–2017 and case density changes in 2006 and 2017 over age of DS-TB **(A,D)**, INH/RFP-resistant TB **(B,E)**, and MDR-TB **(C,F)**. In **(D,E)**, red dashed-and-dotted and green solid lines represent densities in 2006 and 2017, respectively. We show the changes in case *proportions* of young adults (18–35) and seniors (60+) in 2006 and 2017 in the top-right corner for males and in the bottom-right corner for females; the corresponding P-Values for the decrease in case proportion for young adults and the increase in that for seniors between 2006 and 2017 *via* One-sided Two-Proportion Z Tests are also demonstrated, respectively.

Figure 5 compares the historical trends of drug-sensitive (DS)-TB and DR-TB from 2006 to 2017. Both DR-TB and MDR-TB witnessed an increasing trend. There were significant *proportion* shifts for females from young adults to seniors within both DR-TB and MDR-TB cases. These results were consistent with those in Figure 2.

**Figure 6:**
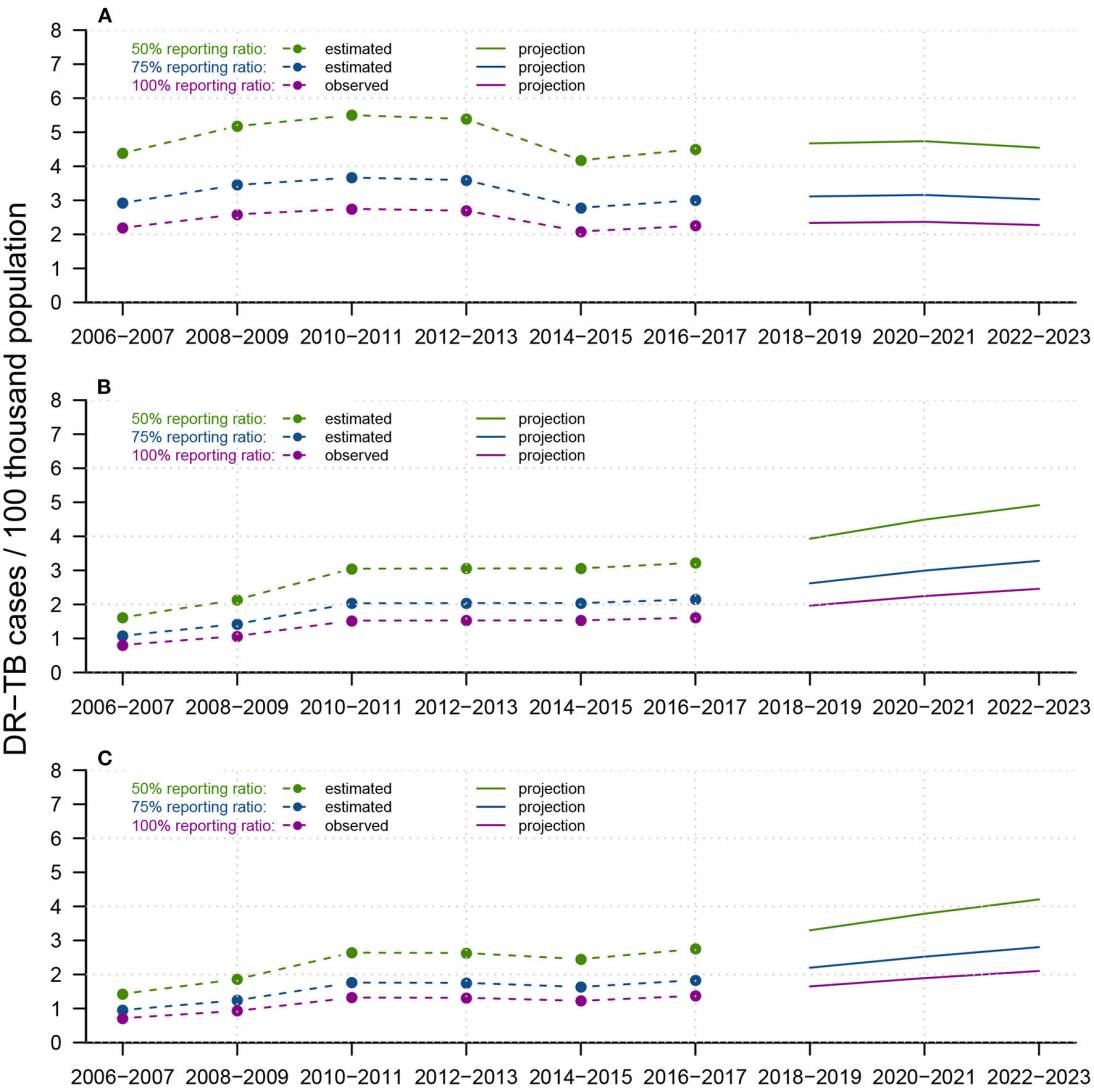
Reported trends from 2006-2017 and forecasts from 2018-2023 of DR-TB infections for **(A)** INH-resistant TB, **(B)** RFP-resistant TB, and **(C)** MDR-TB, among the total population in Shandong, China. Within each panel, dotted-and-dashed lines and solid lines colored in green/blue/purple indicate reported DR-TB infections and forecast trends, under assumed test ratios of 50/75/100%, respectively. We constructed APC models to predict future total DR-TB notifications and smoothed splines to predict the future population in Shandong.

In Figure 6, by assuming three different reporting ratios (i.e.,50, 75, and 100%), we described three possible projections for INH-resistant, RFP-resistant and MDR cases, respectively, from 2018–2023. The historical trend of INH-resistant cases experienced slight growth from 2006–2011 and a sudden drop in 2014–2015 before another small increase. We predicted a gradual decrease from 2018-2023, and there were supposed to be approximately 2–4 INH-resistant cases per 100 thousand population in Shandong by the end of 2023. For both RFP-resistant and MDR cases, a relatively sharp increase emerged from 2006–2009, followed by a long-term plateau from 2010–2017, where DR-TB cases per 100 thousand population remained between 1–3 annually. However, projections showed strong increases after 2018, with RFP-resistant cases per 100 thousand population at approximately 2–4, and MDR cases per 100 thousand population at approximately 1.8–3.5 by the end of 2023. Figure 6 suggests that before 2016–2017, INH-resistant cases were the primary source of MDR-TB cases, but RFP-resistant cases were predicted to show substantial increases in the future and become the dominant component of MDR-TB cases.

## 4. Discussion

TB has been one of the most onerous burdens on public health in Shandong, China. Under the guidance of the “Twelfth Five-Year Plan” for TB control proposed in 2012, province- and city-level governments have improved surveillance, screening, and control strategies with remarkable results (27). Jinan, Yantai, Linyi, Liaocheng, Weifang, Jinning, and Dezhou ranked 1st, 2nd, and 5th–9th in population out of 17 cities in the province, in which Jinan is the capital city, in 2010 (12). By studying the heterogeneity in TB incidence rates in these cities, our results not only revealed heterogeneities between cities, sex, and age groups for TB and DR-TB notifications but also provided crude forecasts, insights for the affected population, and TB and DR-TB control and prevention strategies. A large number of migrants and the aging population are two serious barriers to the further prevention of and reduction in TB incidence (10, 28–31). Therefore, our research has shown the underlying relationship between these two factors and the heterogeneity in TB incidence factors.

With significant proportions of rural populations, Jining (42.9%), Linyi (42.6%), Dezhou (44.43%) and Liaocheng (49.66%) had the most TB notifications among the seven cities, whereas Jinan (29.47%), Yantai (36.34%) and Weifang (40.05%) had the least (12). The rapid progression of urbanization provides better medical facilities in these regions, while simultaneously leading to a significant increase in the migrant population from rural areas to urban areas. Due to a lack of proper screening and prevention measures, dramatic increases emerged in TB notification rates among migrants; these lack of measures will make further reductions in TB incidence rates in cities such as Linyi and Liaocheng much more difficult.

Compared to transmitted TB, which usually occurs among young adults, reactivated TB usually occurs among the senior population (7) and requires much more effort for detection, prevention, and treatment. Along with the “aging” population, increasing proportion of TB cases from seniors among females from 2006–2017 is undoubtedly responsible for sustaining and speeding up the decline in TB incidence in China. Our forecasts for both TB notifications and DR-TB notifications *via* APC models further validate this concern.

Due to urbanization across China, many migrant farmers migrated to the cities and thus became the primary source of TB infections, consistent with previous studies in China (32). Screening and prevention are priorities for migrant farmers and can help reduce the TB notification rates among migrants, further reducing the spread of TB in cities. In addition, measures including prevention education, regular surveillance and diagnostic awareness improvement can be useful as well. College students are the second primary source of TB infections (33) in Shandong, especially in Jinan and Yantai; this occurs because there are more colleges and universities in Jinan (46) and Yantai (17) than in Weifang (9), Jining (9), Linyi (6), Dezhou (5), and Liaocheng (4). A high population density on campus and frequent interactions between students increase the prevalence of transmitted TB among young adults. TB control measures and treatment programs in China (11) have effectively reduced the number of transmitted TB infections among young adults. The TB notification rates differed notably between males and females from 2006–2017. In addition to differential behaviors, such as contact patterns and diagnosis delays (32, 34), urbanization and aging, which prolongs life expectancy and increases the risk of TB reactivation, may also play an important role. Consistent with previous studies that suggested a focus on the population aged over 65 years for vaccine implementations (35), we further demonstrated that senior females are the top priority to continue the decline in TB incidence in China.

In general, DR-TB notifications in Shandong have experienced a gradual increase over the past decade and are estimated to grow continuously in the coming six years. We propose that RFP-resistant notifications will replace INH-resistant notifications to become the primary source of MDR-TB notifications. Technical improvements in DR-TB detection, increasing reporting ratios and extended transmission of DR-TB strains (36, 37) may partially explain this change. Furthermore, we suggest that increasing proportions of reactivated TB among senior females and among the aging population should be a primary concern. In addition to improving surveillance systems and treatment techniques, drug choice for the treatment of drug-resistant TB is another crucial factor. Because EMB-resistant TB has maintained a low correlation with both INH and RFP, which makes EMB a better choice than SM for use in treating MDR-infected patients (Supplementary Figure S11).

There were several limitations in our study: (i) population data and DR-TB data were far from sufficient to elucidate complete and precise inferences and predictions; (ii) age and period effects were not entirely differentiated in our 15-year TB notification data; (iii) the forecasting of future trends relies heavily on the population prediction for TB notification and that of future DR-TB records; therefore, different predicting methods may lead to diverse future trends; and (iv) the rate of DR-TB notifications can be affected by new testing technologies, such as Gene Xpert, which our model did not consider.

In conclusion, our results highlight the heterogeneities among sex, age groups, regions, and occupations for TB notifications, as well as a continuing increase in MDR-TB notifications in the future. Based on criteria such as rural population size, occupation, and sex, we suggest varied focuses for the future control of TB: (i) raising awareness among college students in urban cities, such as Jinan, while improving medical facilities in rural regions, such as Linyi and Liaocheng; (ii) regular surveillance of the senior population, especially senior females; and (iii) strengthening detection techniques for reactivated TB over the next decade.

## Data Availability Statement

The data analyzed in this study is subject to the following licenses/restrictions: the data is confidential and was retrieved from the Tuberculosis Information Management System, the Chinese Center for Disease Control and Prevention (CDC) and the TB Surveillance System. Requests to access should be directed to HL, lihuaichen@163.com.

## Author Contributions

HL and DH: designed the study. QL, SS, AC, SZ, and DH: conducted the literature search. HL, YLiu, CY, NT, YLi, and YS: collected the population data and TB notification data. QL, SS, SZ, and DH: conducted descriptive and statistical data analysis and data visualization. QL, SS, SZ, DH, and HL: conducted data interpretation. QL, AC, and DH: wrote the first draft. QL, SS, and DH: finished the final draft of the article. All authors contributed to the article and approved the submitted version.

## Funding

The work was supported by Key Research and Development Program of Shandong Province [2017GSF218052], Jinan Science and Technology Bureau (CN) [201704100], and Hong Kong Research Grants Council General Research Fund [15205119].

## Conflict of Interest

The authors declare that the research was conducted in the absence of any commercial or financial relationships that could be construed as a potential conflictof interest.

## Publisher's Note

All claims expressed in this article are solely those of the authors and do not necessarily represent those of their affiliated organizations, or those of the publisher, the editors and the reviewers. Any product that may be evaluated in this article, or claim that may be made by its manufacturer, is not guaranteed or endorsed by the publisher.
