# Supplementary material for: Changing Epidemiology of TB in Shandong, China Driven by Demographic Changes
Source: Front Med (Lausanne). 2022 Mar 9;9:810382. doi: 10.3389/fmed.2022.810382 (PMC8959836; doi:10.3389/fmed.2022.810382)
Supplement: Supplementary file 1 [file Data_Sheet_1.PDF]

frontiers

Supplementary Material

We extracted annual population data from 2006 to 2017 of these seven cites from their Statistics Yearbooks. This dataset contains both total population data, corresponding sex and age structure (i.e., ,0–17, 18–35,35–60, and over 60), and residential population (i.e., people living in the city for over half a year) data. In the meanwhile, we retrieved a detailed age structure (i.e.,0, 1–4, 5–9, 10–14, ···, 95–99, and over 100) for both sexes for the residential population in the same seven cities in 2010 from The Sixth National Population Census. We combined these two sets of population data by applying the detailed age structure from Census in 2010 to data from Yearbooks from 2006 to 2017, constructing a complete demographic statistic from 2006 to 2017 for all seven cities. Eventually, we arrange the TB notifications and demographic data from 2006 to 2017 into 72 age groups, and the DR-TB tested notifications into 34 age groups and seven period groups.

As shown in Table **??**, we identified 22,756 notifications in Jinan, 28,107 in Yantai, 63,089 in Linyi, and 35,376 in Liaocheng. Except for Yantai, which was missing residential information, approximately 90% of TB notifications in the other three cities were from local residents. Young adults (18–35 years old) and seniors (over 60 years old) were the two leading sources for TB notifications among all four cities. Regarding occupation, farmers had the highest TB burden, followed by students, workers and homemakers/unemployed. However, the proportion of farmers varied greatly between Jinan and Yantai (53.48% and 63.55%) and Linyi and Liaocheng (91.91% and 86.98%), as the former two cities had higher urbanization rates (70.53% and 63.66%) (**?**).

To calculate the exact population for each age for either sex in these cities from 2006 to 2017, we conducted the following steps for either sex for each city: (i) Computing the residential population for specified sex and every *rough age group* from 2006 to 2017, using the annual ratio of residential population over total population; (ii) Computing the ratios of population in each year over that in the year of 2010 for each *rough age group*; (iii) Applying the above ratios for *rough age group* in annual population data to the *detailed age group* data in 2010 to get the population for each *detailed age group* from 2006 to 2017; (iv) Subdividing each *detailed age group* to get the population of each age, by considering the proportion of each age is the same within the group.

For investigation on TB notification rate, we first eliminate the notifications from individuals who were over 100 years old, due to negligible TB prevalence among the corresponding population (less than 0.03% overall). The first 15 age groups (*i.e.,* 0–14) and the last 16 age groups (*i.e.,* 85–100) are then aggregated in two ensembles: under 15 years old and over 84 years old. We eventually consolidate the notifications and demographic data from 2006 to 2017, which are divided into 72 age groups, for the APC model to explore the effects of age, birth cohort, and calendar period.

For investigation on DR-TB tested notification rate, notifications with age under 17 (0.3% overall) or over 84 (0.18% overall) are first eliminated. To compensate for the shortness of data insufficiency, which leads to 0 records for certain ages in several years, we then combine the notifications every two years. At the end, INH-resistant notifications, RFP-resistant notifications, and MDR notifications are divided into 34

age groups (*i.e.,* 17–18, 18–19, $\cdots$, 81–82, 83–84) and 7 period groups (*i.e.,* 2004–2005, $\cdots$, 2016–2017), respectively.

We present the estimated effects of age, period and cohort for DR-TB tested notification rate in Fig **??**. In general, the estimated effects for MDR notifications are nearly identical to those for RFP-resistant notifications, as MDR is the intersection of INH-resistant and RFP-resistant notifications. While, all of the three effects for INH-resistant notifications show higher resistant risk than MDR and INH. Among these three intrinsic factor, period effects play the most essential role in DR-TB notifications. The period effects of three types of DR-TB are increasing in a main through years, though two temporary falls occurred in 2006–07 and 2014–15 in INH-resistant notifications. With continuous fluctuations through the whole range, age effects and cohort effects are mostly stable and low, indicating little difference between ages and birth cohorts on contributing the resistant risk.

We included 12,984 DR-TB laboratory records with complete drug sensitivity information in this study (Table **??**). A total of 22.27% (21.5%–23.05%) of cases presented resistance to at least one of the four commonly used drugs among first-diagnosed patients and 30.59% (28.56%–32.63%) of cases among those with previous treatment presented resistance. For all four drugs, the proportion of drug-resistant cases was generally higher among patients with a treatment history than among first-diagnosed patients. Among first-diagnoses, 6.47% (6.01%–6.93%) were MDR-TB, while among cases with previous treatment, 13.8% were MDR-TB. Regarding RFP-resistant, EMB-resistant and MDR cases, the proportions among previously treated cases significantly exceeded those among first-diagnosed cases. However, on the basis of drug-resistant notifications, 80.3% DR-TB cases and 72.4% MDR-TB cases were from first-diagnosed patients, which is consistent with previous studies in Shandong (**???**).

Table S1. Summary of TB notifications across seven cities in Shandong.

	Jinan (N=22 756)		Yantai (N=28 107)		Linyi (N=63 089)		Liaocheng (N=35 376)		Weifang (N=32 020)		Jining (N=35 205)		Dezhou (N=26 642)	
	no.	% (95% CI)	no.	% (95% CI)	no.	% (95% CI)	no.	% (95% CI)	no.	% (95% CI)	no.	% (95% CI)	no.	% (95% CI)
Gender														
Male	15 154	66.59 (65.98–67.21)	20 675	73.56 (73.04–74.07)	45 939	72.82 (72.47–73.16)	23295	65.85 (65.36–66.34)	22 553	70.43 (69.93–70.93)	25 551	72.58 (72.11–73.04)	17 371	65.2 (64.63–65.77)
Female	7602	33.41 (32.79–34.02)	7432	26.44 (25.93–26.96)	17 150	27.18 (26.84–27.53)	12081	34.15 (33.66–34.64)	9467	29.57 (29.07–30.07)	9654	27.42 (26.96–27.89)	9271	34.8 (34.23–35.37)
Identity														
Local	18 894	83.03 (82.54–83.52)	–	–	59 841	94.85 (94.68–95.02)	33645	95.11 (94.88–95.33)	28 065	87.65 (87.29–88.01)	33 753	95.88 (95.67–96.08)	24 615	92.39 (92.07–92.71)
Immigrant	3862	16.97 (16.48–17.46)	–	–	3247	5.15 (4.97–5.32)	1731	4.89 (4.67–5.12)	3955	12.35 (11.99–12.71)	1452	4.12 (3.92–4.33)	2026	7.6 (7.29–7.92)
Age groups														
0–17	594	2.61 (2.4–2.82)	715	2.54 (2.36–2.73)	1186	1.88 (1.77–1.99)	647	1.83 (1.69–1.97)	1177	3.68 (3.47–3.88)	783	2.22 (2.07–2.38)	511	1.92 (1.75–2.08)
18–35	8021	35.25 (34.63–35.87)	10540	37.5 (36.93–38.07)	15922	25.24 (24.9–25.58)	9890	27.96 (27.49–28.42)	11608	36.25 (35.73–36.78)	10441	29.66 (29.18–30.13)	7703	28.91 (28.37–29.46)
36–60	7822	34.37 (33.76–34.99)	10114	35.98 (35.42–36.55)	23981	38.01 (37.63–38.39)	13386	37.84 (37.33–38.34)	11177	34.91 (34.38–35.43)	12926	36.72 (36.21–37.22)	9411	35.32 (34.75–35.9)
over 60	6319	27.77 (27.19–28.35)	6738	23.97 (23.47–24.47)	22000	34.87 (34.5–35.24)	11453	32.38 (31.89–32.86)	8058	25.17 (24.69–25.64)	11055	31.4 (30.92–31.89)	9017	33.85 (33.28–34.41)
Occupations														
Students	2129	9.36 (8.98–9.73)	1851	6.59 (6.3–6.88)	1534	2.43 (2.31–2.55)	1080	3.05 (2.87–3.23)	2720	8.49 (8.19–8.8)	1382	3.93 (3.72–4.13)	807	3.03 (2.82–3.23)
Workers	1698	7.46 (7.12–7.8)	2036	7.24 (6.94–7.55)	733	1.16 (1.08–1.25)	1153	3.26 (3.07–3.44)	2511	7.84 (7.55–8.14)	1302	3.7 (3.5–3.9)	1309	4.91 (4.65–5.17)
Migrant workers	285	1.25 (1.11–1.4)	1002	3.56 (3.35–3.78)	643	1.02 (0.94–1.1)	238	0.67 (0.59–0.76)	338	1.06 (0.94–1.17)	224	0.64 (0.55–0.72)	605	2.27 (2.09–2.45)
Farmers	12 171	53.48 (52.84–54.13)	17 863	63.55 (62.99–64.12)	57 983	91.91 (91.69–92.12)	30 770	86.98 (86.63–87.33)	22 912	71.56 (71.06–72.05)	29 345	83.35 (82.97–83.74)	21 211	79.61 (79.13–80.1)
Household / Unemployed	2635	11.58 (11.16–12)	3418	12.16 (11.78–12.54)	564	0.89 (0.82–0.97)	627	1.77 (1.63–1.91)	812	2.54 (2.36–2.71)	781	2.22 (2.06–2.37)	924	3.47 (3.25–3.69)
Others & unknown	3838	16.87 (16.38–17.35)	1937	6.89 (6.6–7.19)	1632	2.59 (2.46–2.71)	1508	4.26 (4.05–4.47)	2727	8.52 (8.21–8.82)	2171	6.17 (5.92–6.42)	1786	6.7 (6.4–7)

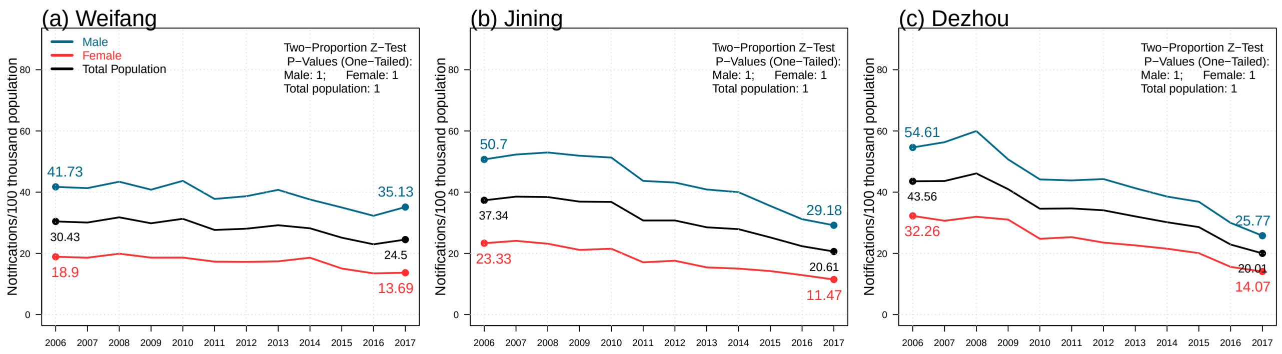

Figure S1. Changes in the notification rates for males, females, and total population from 2006–2017 in (a) Weifang, (b) Jining, and (c) Dezhou. In each panel, the blue, red, and black lines represent notification rate trends; the P-Values of the Two-Proportion Z-Test for males, females, and total population in each cities are shown in the top-right corner.

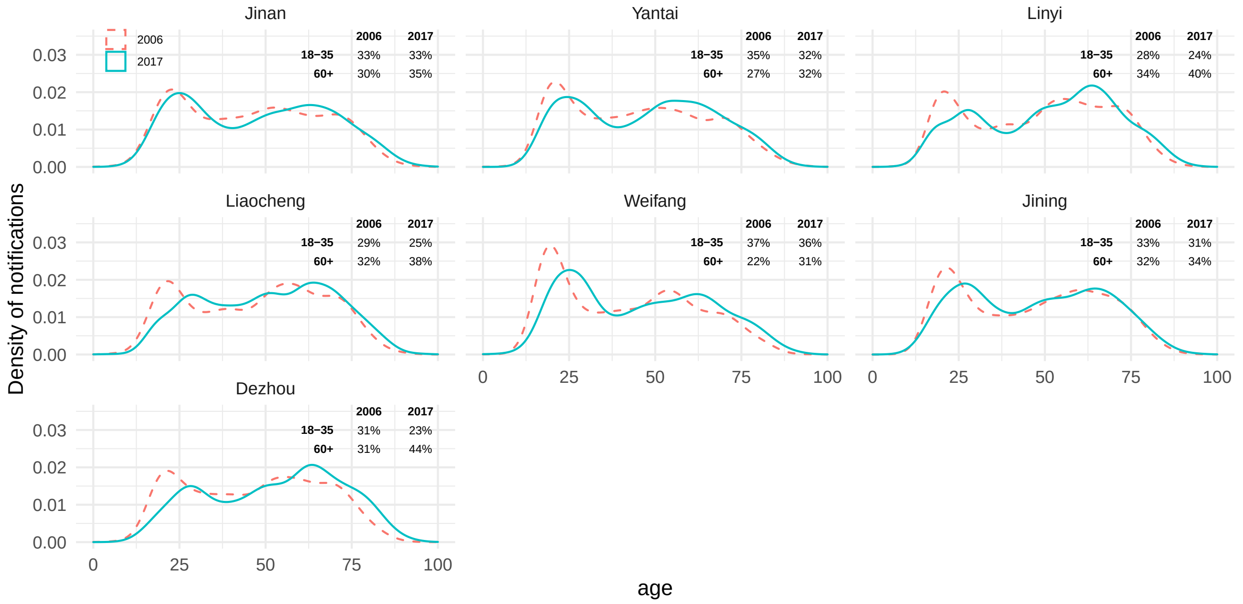

Figure S2. Case density changes in 2006 and 2017 in 7 cities in Shandong, China. In each panel, red and green area represent density in 2006 and 2017, respectively. The top right corner shows the changes of case proportions for young adults (18–35) and seniors (60+) in 2006 and 2017.

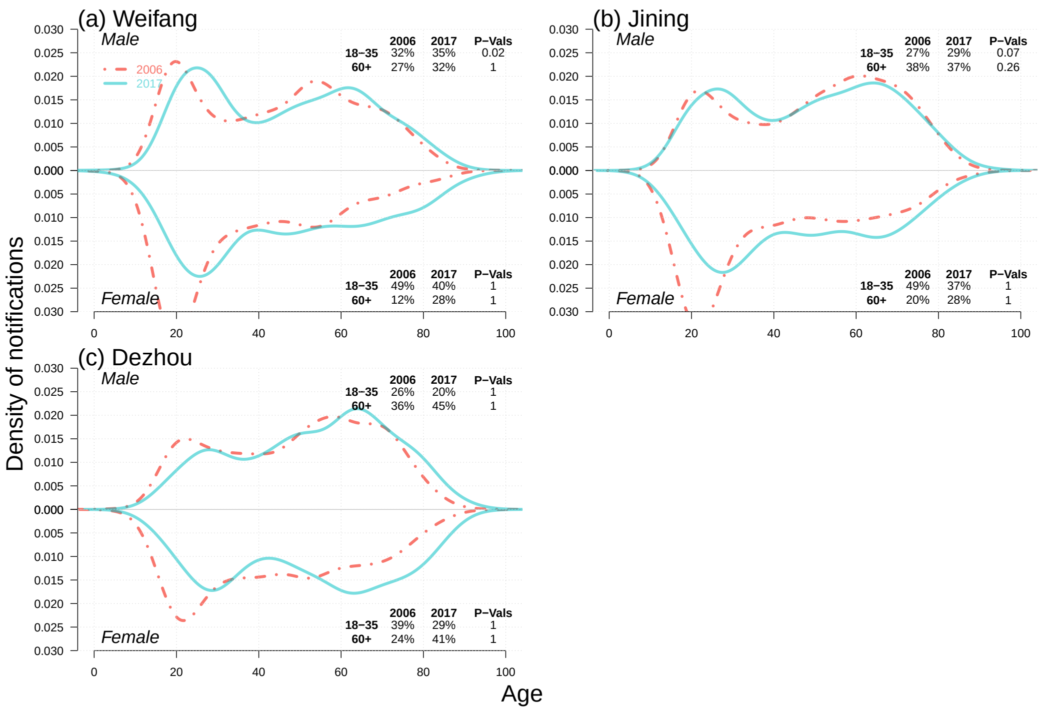

Figure S3. Case density changes in 2006 and 2017 for both male (left column) and female (right column) in 7 cities in Shandong, China. In each panel, red and green area represent density in 2006 and 2017, respectively. The top right corner shows the changes of case proportions for young adults (18–35) and seniors (60+) in 2006 and 2017.

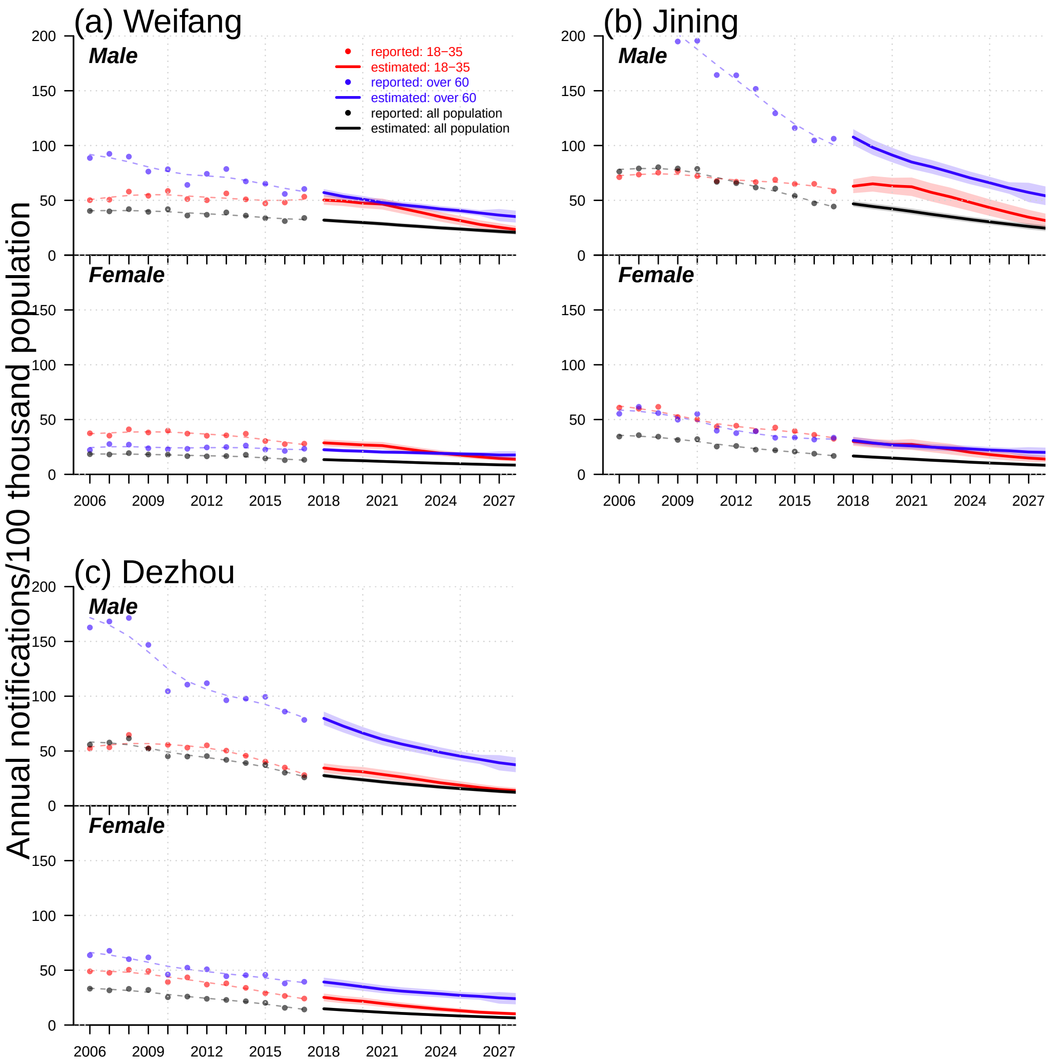

Figure S4. Trends of TB notification rate from 2006 to 2017 and forecast from 2018 to 2027 in three cities in Shandong, China. From top to bottom, each row show TB notification trends of (a) male, and (b) female from left to right, for each city (i.e.,Weifang, Jining, and Dezhou), respectively. Within each panel, we use dots, lines, and shades colored in red/blue/black to indicate reported trends, forecast trends, and 95% confidential interval for forecasts of annual TB notifications (in 100K population) for population under 40 years old/over 40 years old/total population, respectively. Dashed lines colored in red/blue/black are smoothed splines for indicating the trends of annual reported TB notifications for population under 40 years old/over 40 years old/total population, respectively.

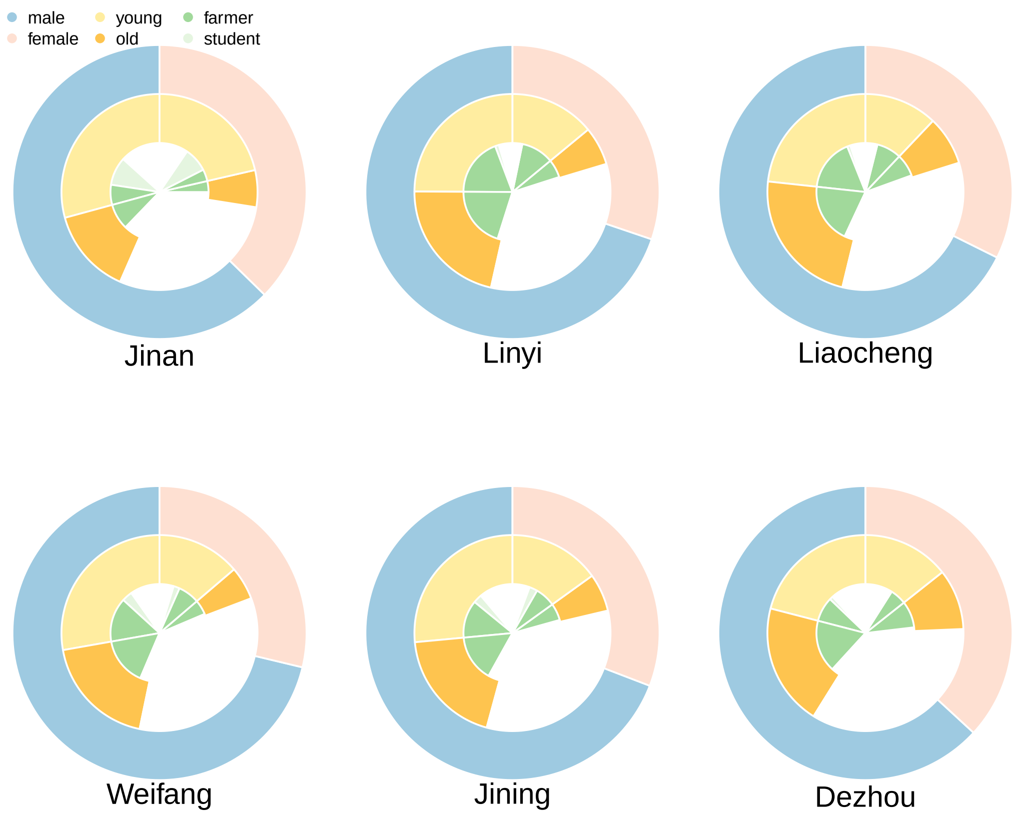

Figure S5. The components of migrant TB notifications.

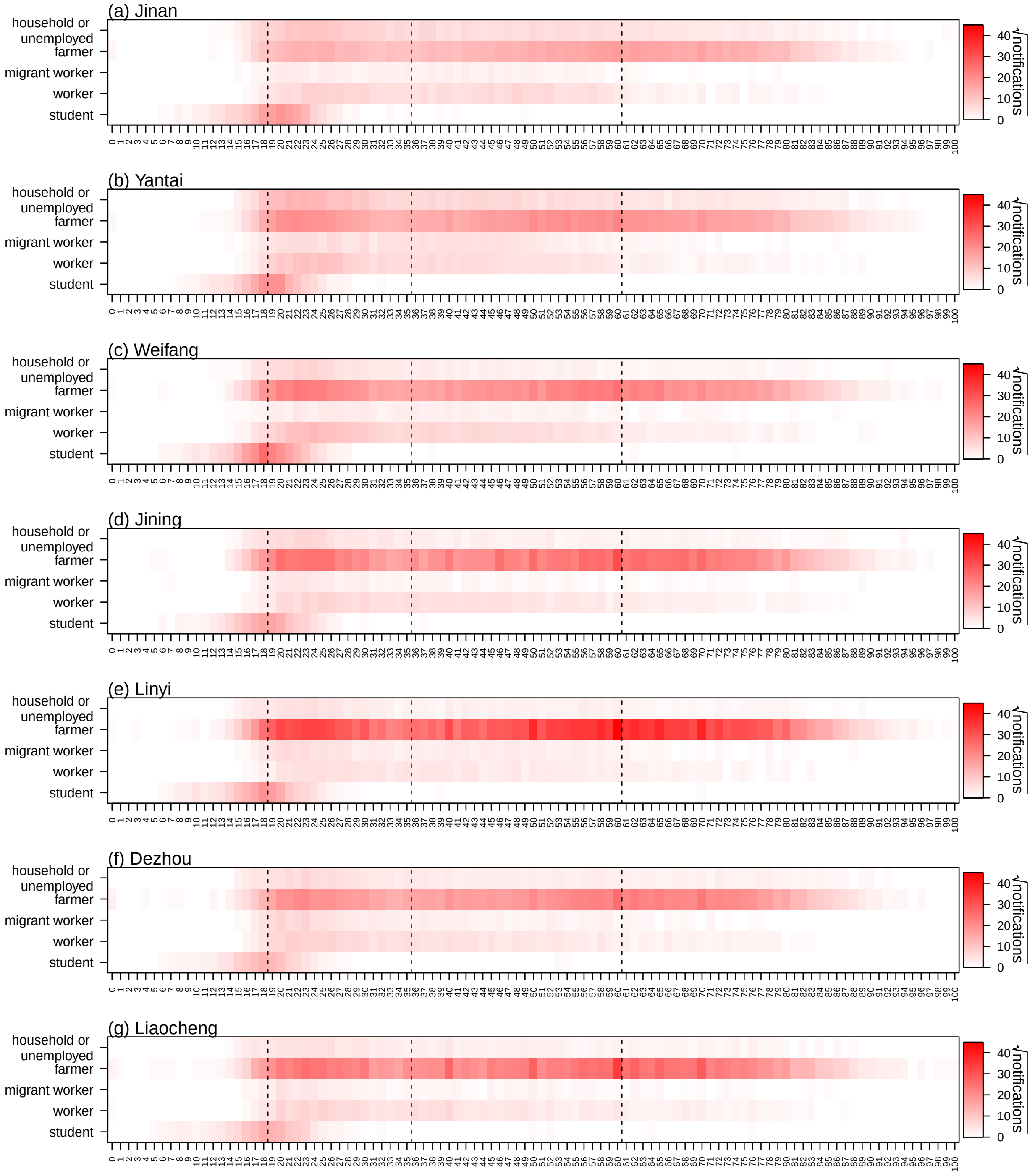

Figure S6. TB notifications against age from 2006 to 2017 for top five occupations in seven cities, from 2006 to 2017: student, worker, migrant worker, farmer and household or unemployed. Dashed-black lines divide the heatmaps into four sub-age groups: 0–17, 18–35, 35–60, and over 60.

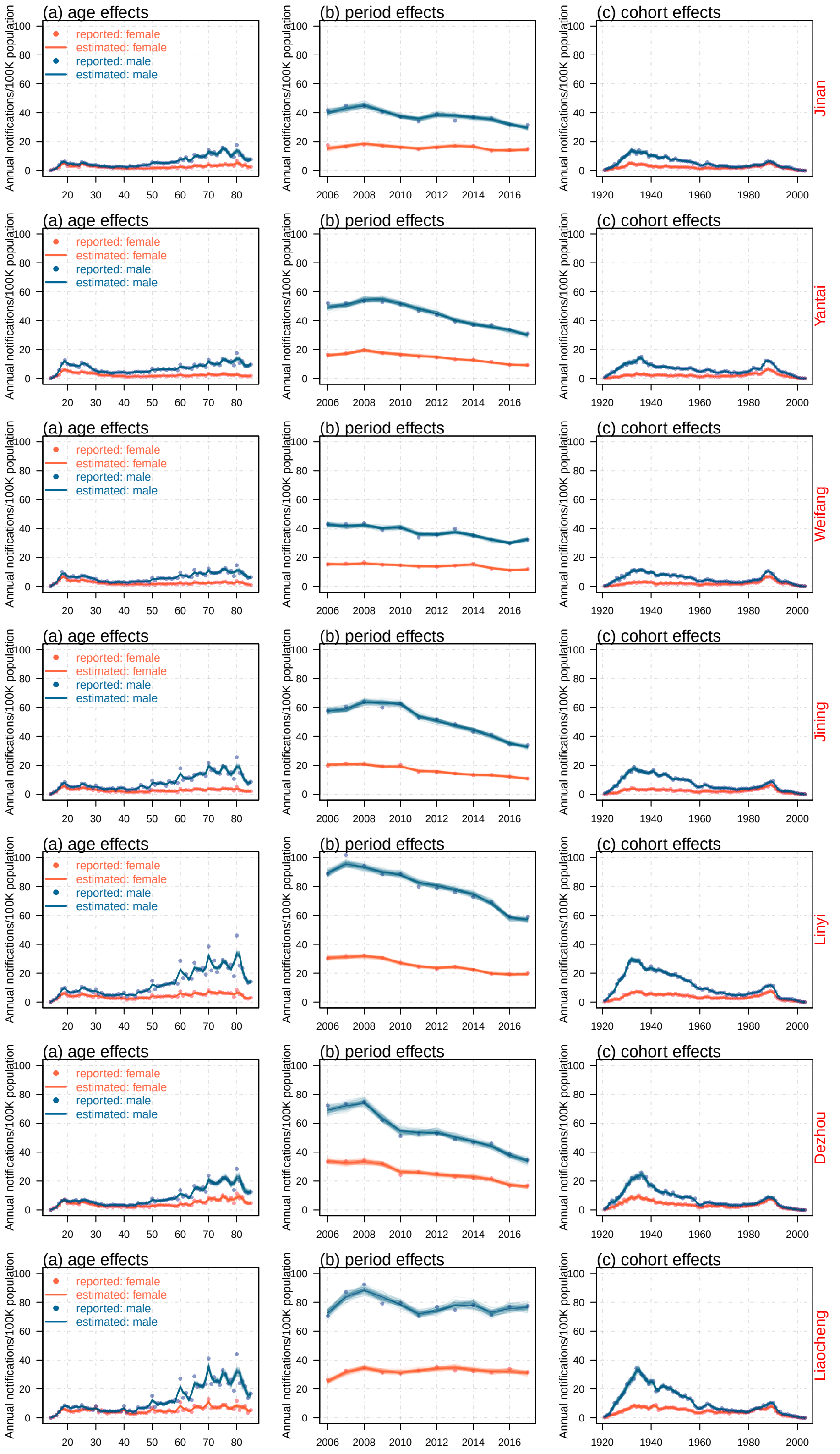

Figure S7. Estimated effects of age, calendar period and birth cohort on TB notification rate from 2006 to 2017 in seven cities in Shandong, China. From top to bottom, each row show (a) age effects, (b) period effects, and (c) cohort effects from left to right, for each city (*i.e.*, Jinan, Yantai, Weifang, Jining, Linyi, and Dezhou), respectively. Within each panels, dots, lines, and transparent lines colored in blue/red show reported effects, estimated effects, and bootstrap simulations for estimated effects on annual TB notifications (per 100K population), for male/female, respectively.

Table S2. Summary of drug sensitivity and resistance to four antitubercolosis drugs in Shandong.

Sensitivity or Resistance	First-diagnosed TB notifications (N=11 013)		Previously Treated TB notifications (N=1971)	
	no.	% (95% CI)	no.	% (95% CI)
Sensitivity to all four drugs	8560	77.72 (76.95–78.50)	1368	69.40 (67.37–71.44)
Resistance to four drugs				
At least one of four drugs	2453	22.27 (21.5–23.05)	603	30.59 (28.56–32.63)
INH	1628	14.78 (14.12–15.45)	450	22.83 (20.98–24.68)
RFP	837	7.60 (7.11–8.10)	326	16.54 (14.90–18.18)
EMB	365	3.31 (2.98–3.65)	124	6.29 (5.22–7.36)
SM	1808	16.41 (15.73–17.11)	438	22.22 (20.39–24.06)
INH or RFP (but not both)	1039	9.43 (8.89–9.98)	232	11.77 (10.35–13.19)
MDR	713	6.47 (6.01–6.93)	272	13.80 (12.28–15.32)

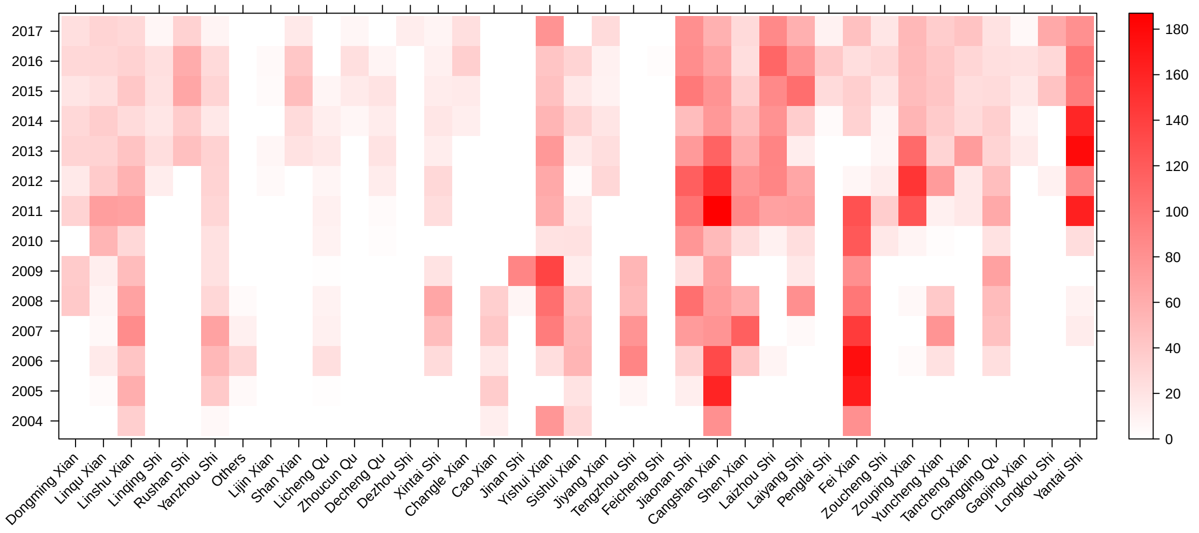

Figure S8. Number of DR-TB notifications across 38 units in 37 regions in Shandong, from 2004 to 2017.

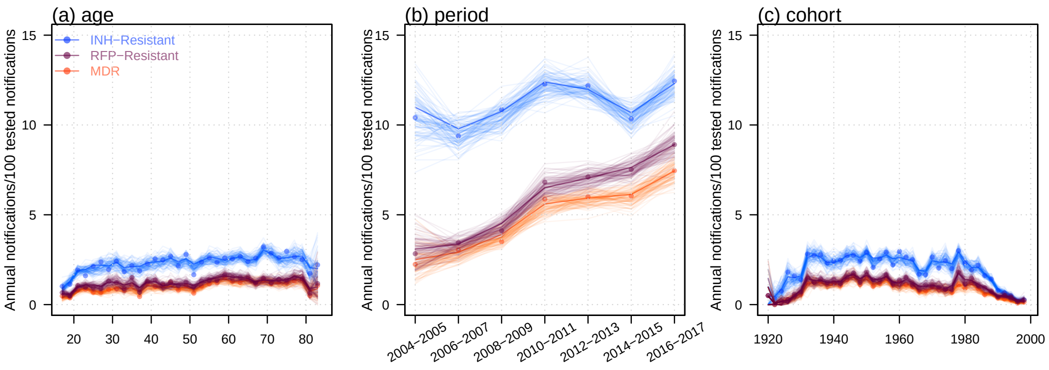

Figure S9. Estimated effects of (a) age, (b) calendar period and (c) birth cohort on DR-TB tested notification rate from 2004 to 2017 in Shandong, China. Within each panel, dots, lines, and transparent lines colored in blue/brown/orange show reported effects, estimated effects, and bootstrap simulations for estimated effects on annual TB notifications (per 100K population), for INH-resistant TB/RFP-resistant TB/MDR-TB, respectively.

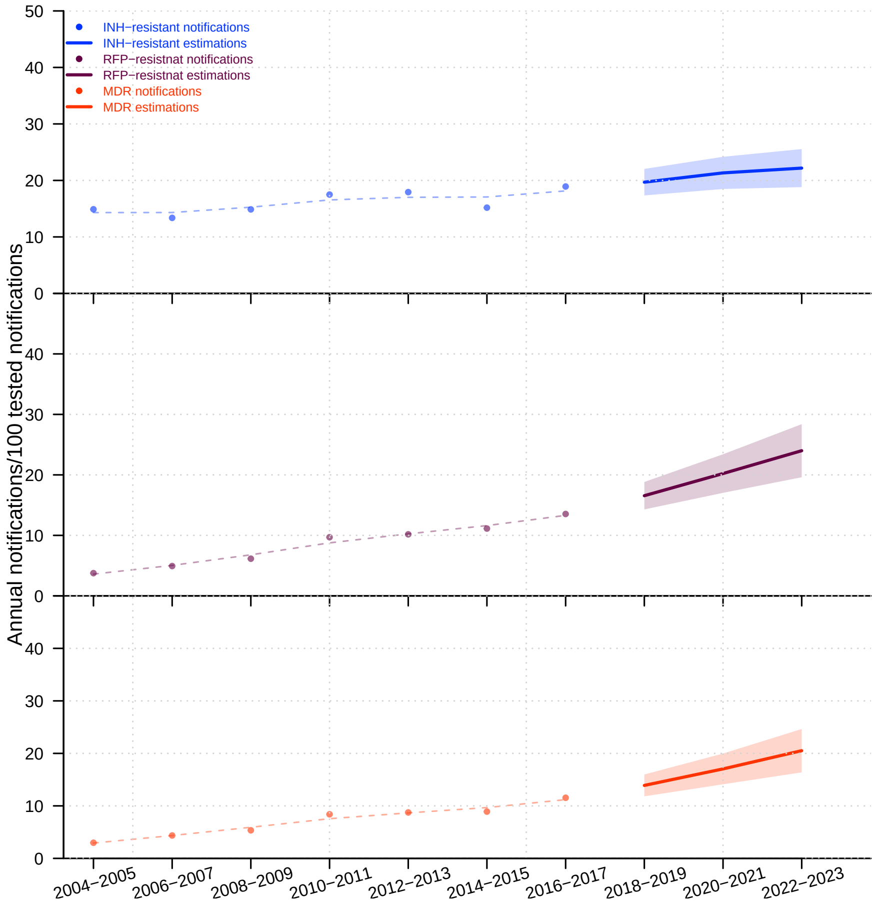

Figure S10. Reported trends from 2004 to 2017 and forecasts from 2018 to 2023 of DR-TB tested notification rate in Shandong, China for INH-resistant TB, RFP-resistant TB, and MDR-TB, from top to bottom, respectively. Dots, lines, and shaded area colored in blue/brown/orange show reported tested notification rates (per 100 tested notifications), estimated tested notification rates, and 95% confidence interval for estimation, for INH-resistant TB/RFP-resistant TB/MDR-TB, respectively. Dashed lines colored in blue/brown/orange are smoothed splines for indicating the trends of reported DR-TB tested notification rate for INH-resistant TB/RFP-resistant TB/MDR-TB, respectively. Future total collected DR-TB records is predicted by APC models.

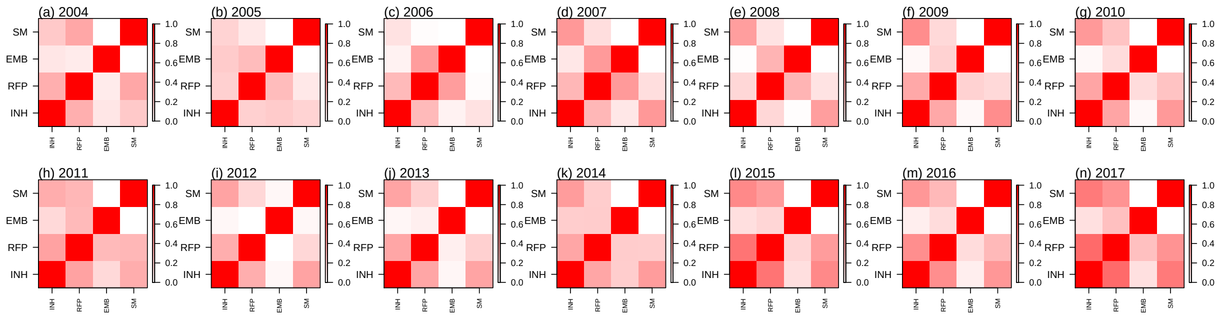

Figure S11. Correlation between four types of commonly used drugs from 2004 to 2017. Darker color indicates higher correlation.
